# Thymidine phosphorylase activates NFκB and stimulates the expression of angiogenic and metastatic factors in human cancer cells

**DOI:** 10.18632/oncotarget.2242

**Published:** 2014-10-31

**Authors:** Sho Tabata, Ryuji Ikeda, Masatatsu Yamamoto, Shunji Shimaoka, Naofumi Mukaida, Yasuo Takeda, Katsushi Yamada, Tomoyoshi Soga, Tatsuhiko Furukawa, Shin-ichi Akiyama

**Affiliations:** ^1^ Institute for Advanced Biosciences, Keio University, Kakuganji, Tsuruoka, Yamagata 997-0052, Japan; ^2^ Department of Clinical Pharmacy and Pharmacology, Graduate School Medical and Dental Science, Kagoshima University, Kagoshima 890-8544, Japan; ^3^ Department of Molecular Oncology, Graduate School Medical and Dental Science, Kagoshima University, Kagoshima 890-8544, Japan; ^4^ Department of Gastroenterology, Nanpuh Hospital, Kagoshima 892-0854, Japan; ^5^ Department of Molecular Oncology, Cancer Research Institute, Kanazawa University, Kanazawa 920-0934, Japan; ^6^ Department of Clinical Pharmacology, Faculty of Pharmaceutical Sciences, Nagasaki International University, Sasebo Nagasaki 859-3298, Japan; ^7^ Clinical Research Center, National Kyushu Cancer Center, Notame Minami-ku, Fukuoka 811-1395, Japan

**Keywords:** thymidine phosphorylase, IL-8, ROS, NFκB

## Abstract

Thymidine phosphorylase (TP) promotes angiogenesis and metastasis, and confers resistance to anticancer agents in some cancer cell types. We previously reported that TP stimulates the expression of interleukin (IL)-8 in human KB cancer cells by an unknown mechanism. A mutation in the nuclear factor (NF)κB binding site of the *IL-8* promoter suppressed promoter activity in KB/TP cells that overexpress TP. Specifically inhibiting NFκB by using BY11-7082 also suppressed TP-induced *IL-8* promoter activity and *IL-8* expression. Moreover, TP overexpression led to the activation of NFκB and an upregulation in the expression of its target genes, and increased phosphorylated IKKα/β protein levels, while promoting IκBα degradation as well as p65 phosphorylation and nuclear localization. The activation of NFκB in KB/TP cells was suppressed by the antioxidants N-acetylcysteine and EUK-8. In addition, in gastric cancer tissue samples, the expression of the NFκB-regulated genes, including *IL-8*, *IL-6*, and *fibronectin-1* was positively correlated with TP expression. These findings indicate that reactive oxygen species mediated NFκB activation by TP increases the expression of genes that promote angiogenesis and metastasis in gastric cancer.

## INTRODUCTION

Thymidine phosphorylase (TP), also known as platelet-derived endothelial cell growth factor (PD-ECGF), acts as a potent angiogenic factor in tumours [1, 2]. Many types of solid tumour express TP, and high TP activities are correlated with microvessel densities [2]. In addition to angiogenesis, TP promotes tumour invasion and metastasis, and confers resistance to some cancer drugs [3-5]. TP catalyses the reversible phosphorolysis of thymidine and other pyrimidine 2′-deoxyribonucleosides. The conversion of thymidine to thymine and 2-deoxy-d-ribose-1-phosphate generates the dephosphorylated product 2-deoxy-d-ribose [6], which mediates many of the biological activities of TP [3, 4]. We previously reported that TP and 2-deoxy-d-ribose enhanced the expression levels of *interleukin* (*IL*)*-8* mRNA and protein in human KB cancer cells [7].

IL-8 was originally discovered as a chemotactic factor for leukocytes. This chemokine has a CXC amino acid motif and has been shown to contribute to human cancer progression through its potential mitogenic, angiogenic, and motogenic activities. [8] IL-8 expression is also influenced by various conditions in the tumour microenvironment, such as hypoxia, acidosis, nitric oxide level, and cell density [8]. The region between −133 and −69 of *IL-8* 5′UTR promoter is thought to be essential for constitutive activity, containing binding sites for the transcription factors activator protein (AP)-1, nuclear factor (NF)κB, and nuclear factor for interleukin 6 (NF-IL6) among others, which play important roles in the regulation of IL-8 expression [9]. Mutations in the NFκB and AP-1 sites abolish constitutive promoter activity, while some activity is preserved when the NF-IL6 binding site is mutated [9].

It has been suggested that oxidative stress is induced in TP-overexpressing cells, which leads to the upregulation of vascular endothelial growth factor (VEGF), matrix metalloproteinase (MMP)-1, and IL-8 [10], which are implicated in angiogenesis and tumour cell invasion. We previously demonstrated that TP and 2-deoxy-d-ribose enhance reactive oxygen spices (ROS) generation and consequently increase IL-8 expression via an unknown mechanism [7]. In the present study, the molecular basis for TP-induced activation of the *IL-8* promoter was investigated in a human KB cancer cell line and gastric cancer tissue.

## RESULTS

### Effect of TP on the promoter activity of *IL-8*

**Figure 1:**
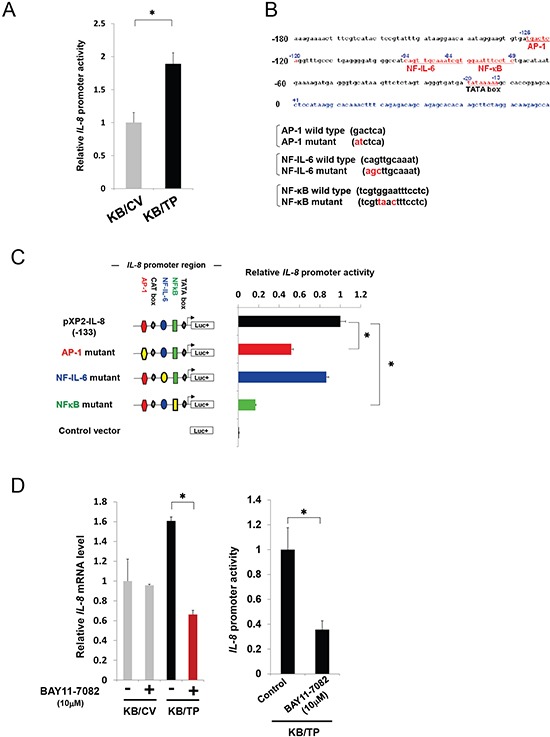
Induction of *IL-8* promoter-driven reporter gene expression **(A)** *IL-8* promoter activity was assessed in KB/TP and KB/CV cells (overexpressing or lacking TP, respectively) by transfecting a construct expressing the luciferase reporter under the control of the *IL-8* promoter. **(B)** Outline of the *IL-8*-promoter constructs used in this study. The binding sites for the transcription factors AP-1, NF-IL6, and NFκB in the promoter region (from −180 bp) upstream of the transcription start site (+1) of the human *IL-8* gene are shown. A plasmid containing the sequence from −133 to +44 (pXP2-IL-8) was used to test *IL-8* promoter activity. **(C)** Plasmids containing mutations in the AP-1 (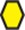), NF-IL6 (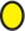), or NFκB (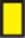) binding site in the human *IL-8* gene promoter were used to drive luciferase (Luc+) expression, and activity was compared to the wild-type promoter. **(D)** Effect of the NFκB inhibitor BAY11-7082 on the transcript expression (left) and promoter activity (right) of *IL-8* in KB cells, as determined by real-time PCR and the luciferase assay, respectively. Data are shown as mean ± SD. *P < 0.01.

We previously demonstrated that IL-8 expression in TP-overexpressing KB/TP cells was higher than in KB/CV that do not express TP [7]. To investigate the mechanism underlying the regulation of *IL-8* expression by TP, *IL-8* promoter activity was assessed by means of a luciferase assay. The activity of the *IL-8* promoter was about 2-fold higher in KB/TP cells than in KB/CV cells (Figure. 1A). These data suggest that *IL-8* expression is mediated by TP-induced promoter activation. The *IL-8* promoter contains binding sites for AP-1 (−126 to −120 bp), NFκB (−80 to −71 bp), and NF-IL6 (−94 to −81 bp; Figure. 1B) [11]. Mutation of the NFκB binding site reduced *IL-8* promoter activity to less than 20% of the wild type value. Mutation of the AP-1 binding site caused a less marked suppression (~50%) of promoter activity, while mutating the NF-IL6 site had no effect (Figure. 1C). Treatment of KB/TP cells with the pharmacological NFκB inhibitor BAY11-7080 also reduced *IL-8* promoter activity (Figure. 1D). These results indicate that the regulation of IL-8 expression by TP is through NFκB.

### Activation of NFκB by TP

**Figure 2:**
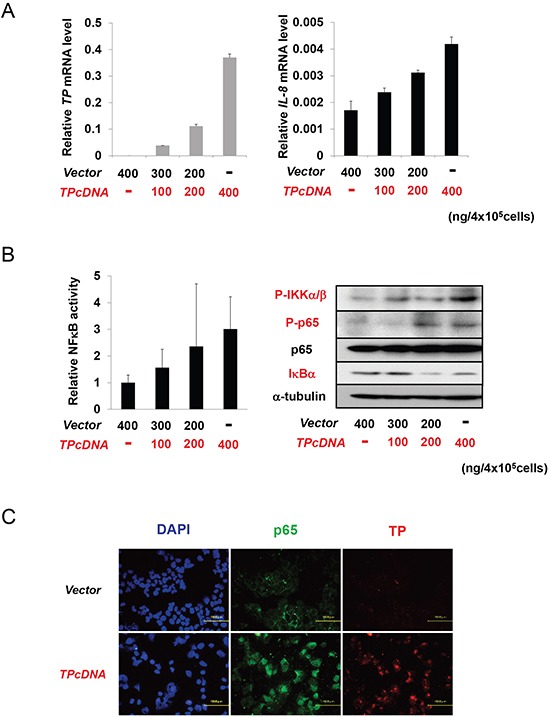
NFκB activation by TP **(A)** *IL-8* transcript expression in KB cells increased with the amount of transfected *TP* cDNA in a dose-dependent manner, as determined by real-time PCR. Cells were transfected with a control plasmid (vector; *i.e.*, KB/CV cells) or indicated amounts of a TP overexpression construct (*i.e.*, KB/TP cells). **(B)** NFκB activity was determined using a luciferase reporter construct (left), and the levels of p-IKKα/β, P-p65, p65, and IκBα were determined by immunoblotting (right), with α-tubulin used as a loading control. **(C)** Effect of TP overexpression on the subcellular localization of p65 in KB cells compared to control cells, as determined by immunocytochemistry; nuclei are stained with DAPI. Scale bar = 100 μm. Data are shown as mean ± SD. *P < 0.01, **P < 0.05.

In canonical NFκB signalling, IKK activation results in IκB phosphorylation and degradation, which shifts the dynamic equilibrium of NFκB cytosolic and nuclear localization in favour of the latter [12]. To explore the effect of TP on the NFκB signalling pathway, KB cells were transiently transfected with *TP* cDNA. *IL-8* transcript levels increased as a function of *TP* cDNA concentration (Figure. 2A). To test whether TP could induce NFκB activation, KB cells were transfected with a luciferase reporter plasmid containing six tandem NFκB binding sites. NFκB activity increased concomitantly with TP expression (Figure. 2B, left). The levels of phosphorylated (P-)IKKα/β and P-p65 were also increased, while IκBα expression was reduced by TP (Figure. 2B, right). The subcellular localization of p65, an NFκB subunit, was examined by confocal microscopy. p65 mainly localized to nuclei of KB cells overexpressing TP, in contrast with a predominantly cytoplasmic localization in control KB cells transfected with the empty vector (Figure. 2C). These findings suggest that TP activates the NFκB signalling pathway and the downstream transcriptional response.

**Figure 3:**
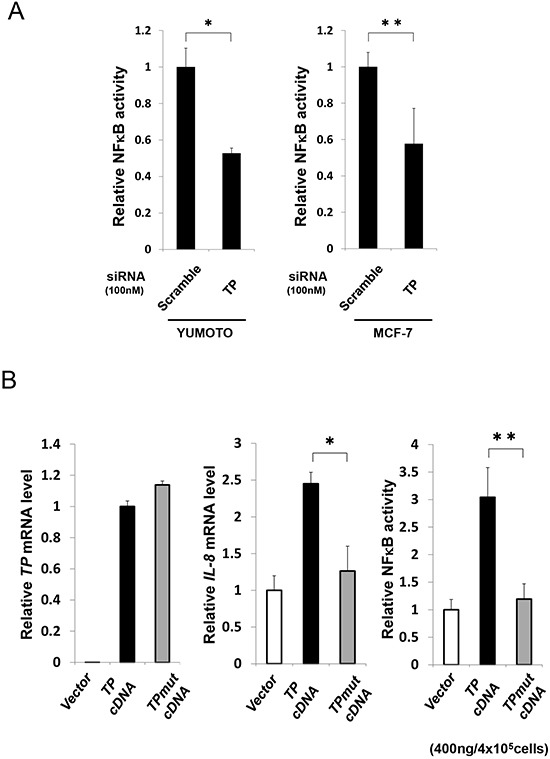
Effect of TP deficiency on NFκB activity **(A)** Yumoto and MCF-7 cells were transfected with control (scrambled) or *TP* siRNA constructs and NFκB activity was determined using a luciferase reporter construct. **(B)** KB cells were transfected with control (vector; *i.e.*, KB/CV cells), or wild-type (TP; *i.e.*, KB/TP cells) or mutant (TPmut; *i.e.*, KB/TPmut cells) TP cDNA. *TP* and *IL-8* transcript levels were determined by real-time PCR, and NFκB activity was determined using a luciferase reporter construct. Data are shown as mean ± SD. *P < 0.01, **P < 0.05.

The effect of TP deficiency on NFκB activation was investigated by siRNA-mediated knockdown of endogenous TP in Yumoto and MCF-7 cells. NFκB activity was reduced in the absence of TP (Figure. 3A). The KB/TPmut cell line expressing a mutant TP lacking phosphorylase function was used to determine whether the enzymatic activity of TP is required for NFκB activation. TP expression was comparable in KB/TPmut and KB/TP cells, and was not detected in KB/CV cells as expected (Figure. 3B). However, IL-8 transcript level and NFκB activity was lower in KB/TPmut than in KB/TP cells (Figure. 3B). These results indicate that the enzymatic activity of TP is required for NFκB-mediated *IL-8* transcription.

### Suppression of TP-induced NFκB activation by antioxidants

**Figure 4:**
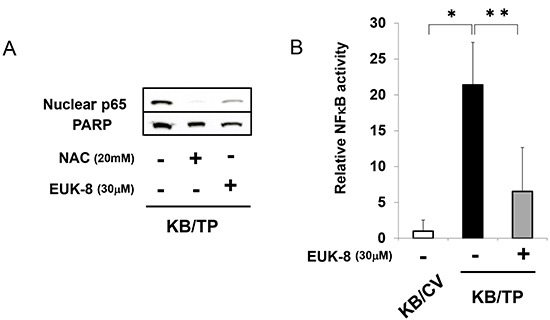
Suppression of TP-induced NFκB activity by antioxidants **(A)** The protein levels of p65 and PARP (loading control) in the nuclear fraction of KB/TP cells was determined after treatment with the antioxidants NAC or EUK-8 by immunoblotting. **(B)** Cells were treated with EUK-8 and NFκB activity was determined using a luciferase reporter construct. Data are shown as mean ± SD. *P < 0.01, **P < 0.05.

We previously reported that ROS generation and IL-8 expression were upregulated in KB/TP cells, but were suppressed in the presence of an antioxidant [7]. The level of p65 in the nuclear fraction was higher in KB/TP than in KB/CV cells, and was decreased by treatment with the antioxidants N-acetylcysteine (NAC) and EUK-8 (Figure. 4A). Moreover, the TP-induced enhancement of NFκB activity was abolished by EUK-8 (Figure. 4B). These findings suggest that TP mediates NFκB-dependent transcriptional response through the production of ROS, which leads to the activation and subsequent nuclear translocation of NFκB.

### TP-induced activation of NFκB target gene expression

**Figure 5:**
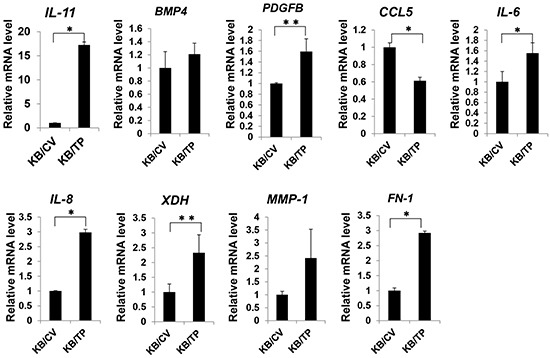
Expression of NFκB target genes Total RNA was extracted from KB/TP and KB/CV cells (overexpressing or lacking TP, respectively), and relative transcript expression levels of the NFκB target genes *IL-11*, *BMP4, PDGFB, CCL5, IL-6, IL-8, XDH, MMP-1*, and *FN-1* were measured using real-time PCR. Each column and bar represents the mean ± SD. *P < 0.01, **P < 0.05.

A microarray analysis was performed in KB/TP cells to determine whether NFκB target genes are upregulated by TP. Of the NFκB-regulated genes examined, 48 had greater than 2-fold higher expression in KB/TP than in KB/CV cells (Supplementary Table S1). Eight of these genes including *IL-8* were implicated in angiogenesis and tumour metastasis. Using real-time PCR, the expression levels of *IL-11*, *platelet-derived growth factor B* (*PDGFB*), *IL-6*, *IL-8*, *xanthine dehydrogenase* (*XDH*), and *fibronectin-1* (*FN-1*) were confirmed as being higher in KB/TP than in KB/CV cells (Figure 5). These results indicate that TP-induced activation of NFκB leads to the upregulation of target genes that promote angiogenesis and metastasis in cancer.

### NFκB activation in TP-positive gastric cancer

**Figure 6:**
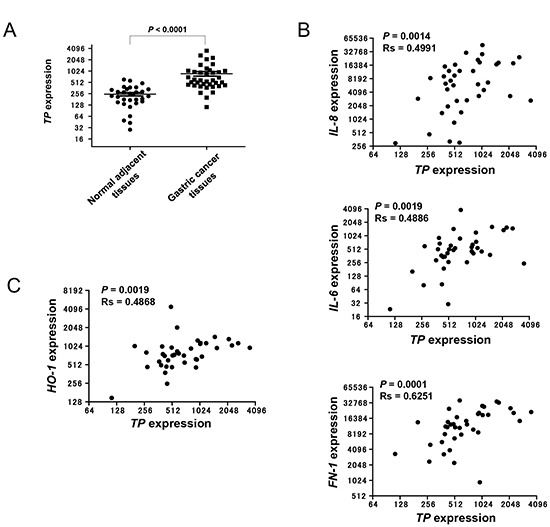
Expression of NFκB target genes in TP-positive gastric cancer tissue **(A)** *TP* mRNA expression levels in gastric cancer tissue samples and normal adjacent mucosa were obtained from the GEO database (see Materials and Methods). **(B)** Correlation of *IL-8*, *IL-6*, and *FN-1* with *TP* expression in gastric cancer tissue samples. **(C)** Correlation of *HO-1*with *TP* expression in gastric cancer tissue samples. Data are shown as mean ± SD. Rs, Spearman's rank correlation coefficient.

Our group has demonstrated that gastric cancer patients who are TP-positive have poor prognoses compared to those who are TP-negative [13]. To determine whether TP activates NFκB in clinical tumours, the D'Errico dataset (GSE13911) from the Gene Expression Omnibus (GEO), which includes the transcriptional profiles of 38 gastric cancer patients [14], was analysed for *TP* expression along with the NFκB target genes *IL-8*, *IL-11*, *bone morphogenetic protein* (*BMP*) *4*, *PDGFB*, *IL-6*, *XDH*, *matrix metalloproteinase* (*MMP*)*-1*, and *FN-1* (Figure 5). The microarray dataset consists of 31-matched normal/tumour tissues and 7-unmatched tumour tissues. TP expression was higher in gastric cancer tissues than in normal adjacent mucosa (Figure. 6A) and was positively correlated with the levels of *IL-8*, *IL-6*, and *FN-1* in tumour tissues (Figure. 6B). Furthermore, we examined whether TP induced the expression of the oxidative stress response gene, *heme oxygenase-1*(*HO-1*) in the D'Errico dataset. *TP* expression significantly correlated with *HO-1* expression in tumours (Figure. 6C).

**Figure 7:**
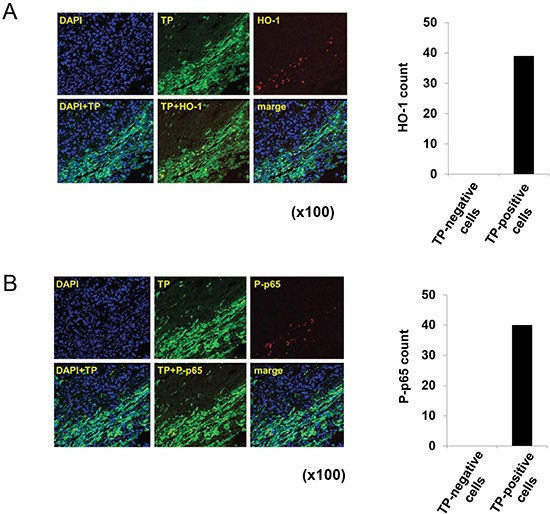
Localization of HO-1 and P-p65 in TP-positive gastric cancer tissue **(A)** Human gastric cancer tissue sample was stained with antibodies against TP and HO-1, and nuclei were stained with DAPI. An overlay (TP+HO-1) shows the localization of HO-1 specifically in TP-positive cells. **(B)** Staining with antibodies against TP and P-p65. An overlay (TP+P-p65) shows the localization of P-p65 specifically in TP-positive cells.

Immunofluorescence labelling of TP-positive gastric tumour tissue samples revealed HO-1 and P-p65 expression in TP-positive, but not in TP-negative cells (Figure. 7A and 7B). Taken together, these findings provide evidence that TP induces oxidative stress and activates NFκB, and consequently stimulates the expression of NFκB target genes that are directly involved in the progression of gastric cancer.

## DISCUSSION

TP expression is upregulated in a variety of solid tumours, which has been positively correlated with microvessel density and poor prognosis [2]. TP is also implicated in tumour angiogenesis and metastasis [2, 4] which, along with tumour growth, can be suppressed by directly inhibiting TP activity [15]. TP catalyses the reversible conversion of thymidine to thymine and 2-deoxy-d-ribose-1-phosphate, which is dephosphorylated to yield 2-deoxy-d-ribose [6], a downstream mediator of TP-induced angiogenesis and metastasis [3, 4]. It has been suggested that the thymidine degradation products 2-deoxy-d-ribose-1-phosphate and 2-deoxy-d-ribose are sources of free radicals, and that this oxidative stress increases the production of the angiogenic factors IL-8, VEGF, and MMP-1 by tumour cells [10]. Our group has reported that thymidine-derived sugars enhance ROS generation, which in turn increases IL-8 expression [7]. However, the mechanisms of induction of angiogenic factors by TP are not fully understood.

We previously found that *IL-8* transcript expression is higher in KB/TP than in KB/CV cells, which do not express detectable levels of TP [4, 7]; this expression was suppressed when cells were treated with TPI, a specific inhibitor of TP enzymatic activity [7]. Accordingly, exposure to the TP substrate thymidine enhanced the expression of *IL-8* in KB/TP but not in KB/CV cells [7]. In the previous study, it was shown that the *IL-8* promoter region contains binding sites for AP-1, NFκB, and NF-IL6, which are presumed to regulate *IL-8* expression [8]. A mutation in the NFκB-binding site attenuated constitutive promoter activity to the greatest extent, and the NFκB inhibitor BAY11-7082 suppressed *IL-8* transcript levels in KB/TP cells (Figure 1). It was also confirmed that the activation of canonical NFκB signalling is higher in KB/TP than in KB/CV cells, providing evidence that TP activates *IL-8* expression via NFκB.

ROS produced by TP activity is required for NFκB activation in KB/TP cells (Figure 4). Previous findings suggest that NFκB activation by H_2_O_2_ is cell-type specific and occurs through different mechanisms [16, 17]; in HeLa cells, H_2_O_2_ induced IKKβ activation, IκBα phosphorylation, and NFκB transcription via protein kinase D, which was activated by two Src-mediated signalling pathways [18]. Since ROS are thought to be generated by 2-deoxy-d-ribose in KB cells, which are derived from HeLa cells [19], it is probable that ROS activates NFκB by a similar pathway in KB cells, although the mechanism of ROS production by TP remains to be elucidated.

In the microarray analysis, several NFκB target genes were upregulated by TP overexpression (Figure 5). Of these, *IL-8*, *IL-6*, and *FN-1* levels were correlated with TP expression in gastric cancer tissues. Most gastric carcinomas express IL-8, and the level is directly correlated with angiogenic activity in the tumour [20]. Overexpression of IL-8 in gastric cancer cells also promotes adhesion, migration, invasion, and chemoresistance [21]. Like IL-8, the expression of IL-6 is higher in gastric cancer than in normal tissues, and is implicated in tumour metastasis [22]. IL-6 was shown to induce cell invasion via activation of c-Src/RhoA/ROCK signalling in a gastric cancer cell line [23]. FN-1 promotes invasion of gastric cancer cells through interaction with α5β1 integrin [24]. These reports are consistent with the idea that TP induce NFκB and the downstream molecules of NFκB confer the malignant phenotype of gastic cancer.

Meanwhile, we have previously reported that gastric cancer overexpresses TP, and the proportion of TP-positive tumours in differentiated adenocarcinomas was higher than that in undifferentiated adenocarcinomas [13]. TP was also expressed mainly in the invasive edges of tumors and was expressed more frequently in macrophage than in tumour cells [13]. Although we showed that TP activated NFκB in gastric cancer tissues, the NFκB activation might occur in tumour-associated macrophages rather than cancer cells. Further work is necessary to examine the TP function in a variety of cell types.

In summary, the present study showed that canonical NFκB signalling was activated by TP possibly as a result of ROS generation, increasing the transcription of *IL-8* and other NFκB target genes. The expression of a subset of these genes—*i.e.*, *IL-8*, *IL-6*, and *FN-1*—along with that of P-p65 was correlated with TP immunoreactivity in gastric cancer tissues. The TP-induced NFκB activation probably plays important roles in angiogenesis and metastasis in gastric cancer, which can inform the development of new drugs for cancer treatment.

## MATERIALS AND METHODS

### Reagents and cell culture

NAC was obtained from Sigma-Aldrich (St. Louis, MO, USA). EUK-8 and BAY11-7082 were obtained from Calbiochem/EMD Millipore (Darmstadt, Germany). Human epidermoid carcinoma KB, cervical carcinoma Yumoto, and breast carcinoma MCF-7 cells were grown in Dulbecco's Modified Eagle's Medium (Nissui Pharmaceuticals Co., Ltd., Tokyo, Japan) containing 10% foetal bovine serum, 2 mM glutamine, and 100 U/ml penicillin at 37°C in a 5% CO_2_ humidified atmosphere. The medium was replaced with serum-free medium before each experiment.

### Stable transfection of PD-ECGF/TP cDNA into KB cells

Plasmids containing the PD-ECGF/TP full-length cDNA, mutant PD-ECGF/TP (L148R9 [25], or the empty vector were transfected into KB cells by electroporation [26]. After Geneticin selection, TP expression in each clone was determined by immunoblotting using an anti-TP monoclonal antibody as described [27]. Positive clones expressing wild-type full-length or mutant TPmut (KB/TP or KB/TPmut cells, respectively) and a clone expressing a control vector without TP expression (KB/CV cells) were used in these experiments.

### Real-time PCR analysis

RNA was extracted from cells using TRIzol reagent (Life Technologies, Gaithersburg, MD) according to manufacturer's instructions, and 1 μg was reverse transcribed using a first-strand cDNA synthesis kit (ReverTra Ace α; Toyobo Co., Ltd., Osaka, Japan). Quantitative real-time PCR was performed using SYBR premix Ex Taq (Takara Bio Inc., Otsu, Japan) on a CFX96 Real-time PCR system (Bio-Rad, Hercules, CA, USA) according to the manufacturers' instructions. Primer sequences are listed in Supplementary Table S2. Quantitation was performed using the ΔΔCt method, with GAPDH expression used as an internal reference. Melt curve analyses confirmed that all real-time PCR products were produced as a single DNA duplex.

### *IL-8* promoter luciferase assay

The region flanking the *IL-8* gene from −133 to +44 bp was subcloned into a luciferase expression vector [28] to yield the pXP2-IL-8 plasmid containing the wild-type promoter. Site-directed mutagenesis of AP-1, NF-IL6, and NFκB binding sites in the *IL-8* promoter was carried out as previously described [29] to obtain mutant promoter constructs (Figure. 1B). KB cells were cotransfected with 1 μg of wild-type or a mutant luciferase plasmid and 0.3 μg of the control plasmid pRL-TK (Promega, Madison, WI, USA) using Lipofectamine 2000 (Life Technologies), and luciferase activity was determined using the Dual-Luciferase Reporter Assay System (Promega) according to the manufacturers' protocols. Luminescence was measured with a luminometer (TD-20/20; Turner Designs, Inc., Sunnyvale, CA, USA). All experiments were performed in triplicate and results were normalized to pRL-TK luciferase activity.

### NFκB activation assay

NFκB activity in KB cells was measured as described [30]. Briefly, KB cells were co-transfected with pBVIx-luc, a luciferase reporter plasmid containing six tandem NFκB binding sites, and pRL-TK, and luminescence was recorded with a luminometer. All experiments were performed in triplicate.

### Immunoblotting

Whole cell extracts were prepared with M-PER reagent (Thermo Fisher Scientific, Waltham, MA, USA) containing phosphatase and protease inhibitor cocktails (Roche Diagnostics, Indianapolis, IN, USA). Protein concentration was measured with Bradford's method, and 50 μg total protein was resolved with on 4–10% NuPAGE Bis-Tris Mini gels (Life Technologies) at 200 V for 40 min, and transferred to a polyvinylidene difluoride membrane (Millipore, Billerica, MA, USA) using the Bio-Rad Transblot SD apparatus [31]. The membrane was treated with Blocking One reagent (Nacalai Tesque, Inc., Kyoto, Japan) for 1 h and incubated with a mouse monoclonal antibody against human TP as previously described [27], or anti-β-actin (Santa Cruz Biotechnology, Inc., Dallas, TX, USA) or anti-α-tubulin antibody (Millipore) overnight at 4°C. After four washes with Tris-buffered saline with 0.1% Triton-X-100 (TBST), the membrane was incubated with HRP-conjugated secondary antibodies (GE Healthcare Bio-Sciences, Pittsburgh, PA, USA) in TBST for 1 h at room temperature. The membrane was then washed and developed using an enhanced chemiluminescence western blotting detection system (GE Healthcare Bio-Sciences). Immunoreactive bands were visualized using a Luminescent Image Analyzer (LAS-4000 mini; Fuji Film, Tokyo, Japan).

### Nuclear protein isolation

Cells were washed with ice-cold phosphate-buffered saline (PBS) and lysed in 400 μl lysis buffer (buffer A) containing 10 mM HEPES [pH 7.9], 10 mM KCl, 0.2 mM EDTA, 1 mM DTT, 0.5 mM PMSF, and 0.6% NP-40. Lysates were centrifuged at 250 × *g* for 10 min. Pellets containing nuclei were washed in buffer A without NP-40 and resuspended in 50 μl nuclear lysis buffer containing 20 mM HEPES (pH 7.9), 0.4 M NaCl, 2 mM EDTA, 1 mM DTT, and 1 mM PMSF, incubated for 30 min at 4°C, and centrifuged at 20,000 × *g* for 20 min. Supernatants were used as the nuclear protein fraction; the nuclear fractions were resolved by polyacrylamide gel electrophoresis and probed for expression of p65 as well as poly ADP ribose polymerase (used as a loading control).

### Immunocytochemistry in cultured cells

Cells cultured on cover slips were washed twice with PBS and fixed with 2% paraformaldehyde for 20 min at room temperature. After washing three times with PBS, cells were permeabilized with 0.3% Triton X-100 in PBS (PBST) for 10 min at room temperature. Cells were blocked with 3% skim milk in PBS for 30 min and incubated for 1 h with an anti-TP or anti-p65 antibody diluted 1:100 in PBS containing 3% skim milk. After washing three times with PBS, cells were incubated for 20 min with Alexa488- or Alexa586-conjugated sheep anti-mouse IgG (Life Technologies) diluted 1:100 in PBS containing 3% skim milk. After three PBS washes, the subcellular localization of TP and p65 was examined using a fluorescence microscope (Olympus, Tokyo, Japan).

### Immunocytochemistry in tissue samples

Gastric tumours were removed surgically at Nanpuh hospital (Kagoshima, Japan) after informed consent had been obtained from the patient. This study was approved by the Ethical Review Board of Kagoshima University Graduate School of Medical and Dental Science and Nanpuh hospital. Samples were fixed with 10% formaldehyde in PBS, embedded in paraffin, and cut into 3-μm thick sections, which were deparaffinized with xylene and dehydrated with 98% ethanol. Endogenous peroxidase was quenched by immersing the slides in 3% hydrogen peroxide in absolute methanol for 10 min at room temperature. Blocked sections were incubated in 10% horse serum in PBST for 30 min at room temperature and at 4°C overnight with primary antibodies. Sections were washed three times in PBS for 5 min and incubated for 30 min with secondary antibodies and the nuclear counterstain DAPI in the dark at room temperature. Images were acquired using a FV500 confocal microscope (Olympus). The primary antibodies were anti-TP (1:1000), anti-P-p65 (1:50) and anti-HO-1 (1:50). For anti-TP, Alexa488-conjugated anti-mouse sheep IgG (1:400) was used as the secondary antibody. For anti-P-p65 and anti-HO-1, Alexa546-conjugated anti-rabbit sheep IgG (1:400) was used as the secondary antibody. In each slide, the number of positive cells was counted under fluorescent microscopy at ×100 (double staining) magnification.

### RNA interference

*TP* and negative control scrambled siRNA duplexes were purchased from Sigma-Aldrich. Cells were transfected with the siRNAs using Lipofectamine 2000 (Life Technologies) according to the manufacturer's instructions.

### Microarray analysis

The cRNA amplified from 500 ng total RNA was labelled using the Quick Amp Labeling Kit, and hybridized to a 44K Agilent 60-mer oligomicroarray (Whole Human Genome Microarray Kit; Agilent Technologies, Inc., Santa Clara, CA, USA) and scanned using an Agilent scanner according to the manufacturer's instructions. Relative hybridization intensity and background hybridization values were calculated using the Agilent Feature Extraction Software (9.5.1.1). Raw signal intensities and Flags for each probe were calculated from hybridization intensities (*i.e.*, Processed Signal) and spot information (*e.g.*, gIsSaturated, etc.), according to the Flag criteria on GeneSpring software (Agilent Technologies, Inc.). A list of genes regulated by NFκB was obtained from nf-κb.org (http://people.bu.edu/gilmore/nf-κb/).

### Accession number

Microarray data were deposited in the National Center for Biotechnology Information GEO with the accession code GSE31467.

### Public microarray data

Public microarray data including the D'Errico dataset (GSE13911) were downloaded from the GEO database. The microarray dataset included the gene expression of 31-matched normal/tumour tissues and 7-unmatched tumour tissues. For subsequent analysis, the dataset transferred to the Subio Platform (Subio, Tokyo, Japan). Mono-channel data in GSE13911 were normalized according to the invariant set method [32]. The gene expression levels were compared using the normalized intensity values.

### Statistical analysis

Data were analysed using GraphPad Prism v5.0 software (La Jolla, CA, USA). In *in vitro* experiments, data for two groups and more than two groups were analysed using the Student's t test and one-way analysis of variance, respectively. Microarray data from GEO were analysed using the Mann-Whitney U test and Spearman's correlation. Data are presented as mean ± SD, and differences were considered statistically significant at *P* < 0.05.

## SUPPLEMENTARY TABLES


