# Supplementary material for: Thymidine phosphorylase activates NFκB and stimulates the expression of angiogenic and metastatic factors in human cancer cells
Source: Oncotarget. 2014 Oct 31;5(21):10473–85. doi: 10.18632/oncotarget.2242 (PMC4279387; doi:10.18632/oncotarget.2242)
Supplement: Supplementary file 1 [file oncotarget-05-10473-s001.pdf]

SUPPLEMENTARY TABLES

Supplementary Table S1. NFκB-regulated genes expressed at higher levels in KB/TP compared with KB/CV.

No.	Gene Symbol	Genbank Accession	*Ratio
**1**	C4BPA	NM_000715	232.834824
**2**	IL11	NM_000641	23.6658529
**3**	TNFSF15	NM_005118	20.1061212
**4**	ABCA1	NM_005502	12.3831103
**5**	BMP4	NM_001202	10.7789127
**6**	KISS1	NM_002256	10.5635615
**7**	EBI3	NM_005755	10.2906536
**8**	DIO2	NM_013989	10.1987009
**9**	ENG	NM_000118	9.51997592
**10**	CXCL10	NM_001565	9.02181919
**11**	CD86	NM_006889	8.19546473
**12**	ADORA1	NM_000674	8.15746054
**13**	ABCC6	NM_001171	8.14736149
**14**	PDGFB	NM_002608	7.7486742
**15**	C3	NM_000064	7.2678143
**16**	CCL5	NM_002985	7.06106394
**17**	SKALP, PI3	NM_002638	7.01803356
**18**	ST6GAL1	NM_173216	6.91645368
**19**	CXCL11	NM_005409	6.68221562
**20**	IL6	NM_000600	6.37254526
**21**	ENO2	NM_001975	5.84153846
**22**	MYB	NM_005375	5.36958059
**23**	GBP1	NM_002053	5.06729041
**24**	FCGRT	NM_004107	4.81283197
**25**	F11R	NM_144503	4.61239598
**26**	IL8	NM_000584	4.54960632
**27**	LAMB2	NM_002292	4.52402276
**28**	ADAM19	NM_023038	4.34813149
**29**	ALOX5	NM_000698	4.09355742
**30**	FN1	NM_212482	3.92905771
**31**	GAD1	NM_000817	3.57324744
**32**	CCR7	NM_001838	3.33221557
**33**	CFB	NM_001710	3.04479532
**34**	TNFSF10	NM_003810	2.98582663
**35**	CCND1	NM_053056	2.84391832

No.	Gene Symbol	Genbank Accession	*Ratio
**36**	XDH	NM_000379	2.74476351
**37**	PSMB9	NM_002800	2.6939827
**38**	CCL1	NM_002981	2.59502851
**39**	CSF1	NM_172212	2.57493128
**40**	CTSB	NM_147780	2.48471773
**41**	NLRP2	NM_017852	2.45039592
**42**	TGM2	NM_004613	2.39482317
**43**	IFNB1	NM_002176	2.37543914
**44**	PTGES	NM_004878	2.33829367
**45**	MMP1	NM_002421	2.28658125
**46**	DPYD	NM_000110	2.14380168
**47**	PTGIS	NM_000961	2.03446736
**48**	TNFSF13B	NM_006573	2.01208417

*Expression level of each gene in KB/TP was divided by that in KB/CV.

Supplementary Table S2. Primers sequences of real-time PCR

Gene Name	Forward Primer	Reverse Primer
TP	5'-GCTGGAGTCTATTCCTGGATTC-3'	5'-ACTGAGAATGGAGGCTGTGATG-3'
IL-8	5'-CCTGATTTCTGCAGCTCTGTGT-3'	5'-GGTGGAAAGGTTTGGAGTATGTCT-3'
GAPDH	5'-GTCAACGGATTTGGTCGTAT-3'	5'-TGGTGATGGGATTTCCATTG-3'
IL11	5'-GACAAATTCCCAGCTGACG-3'	5'-GTAGGACAGTAGGTCCGC-3'
BMP4	5'-CTACATGCGGGATCTTTACC-3'	5'-TGGGATGTTCTCCAGATGTT-3'
PDGFB	5'-ATTCCCGAGGAGCTTTATGA-3'	5'-GTCATGTTCAGGTCCAACTC -3'
FN1	5'-GAATAAGCTGTACCATCGCA-3'	5'-GGTGTCACCAATCTTGTAGG-3'
CCL5	5'-CTGTCATCCTCATTGCTACTG-3'	5'-GCCACTGGTGTAGAAATACTC-3'
MMP-1	5'-GCATCCAAGCCATATATGGAC-3'	5'-GTATCCGTGTAGCACATTCTG-3'
IL-6	5'-CCTCTTCAGAACGAATTGAC-3'	5'-AGTCTCCTCATTGAATCCAG-3'
XDH	5'-GGAACGAGCTTTATTCCACATGGAC-3'	5'-GATACTGAGAGCTTGCATGGCATTC-3'
